# Fracture Resistance of Posterior Milled Nanoceramic Crowns after Thermomechanical Aging

**DOI:** 10.3390/jfb15070171

**Published:** 2024-06-22

**Authors:** Fajer Abdulaziz Alnajjar, Arwa Jamal Alloughani, Mohammed Nasser Alhajj, Mirza Rustum Baig

**Affiliations:** 1 College of Dentistry, Kuwait University, Kuwait P.O. Box 24923, Kuwait; fajer.alnajjar@hscd.ku.edu.kw (F.A.A.); arwaa.loughani@hscd.ku.edu.kw (A.J.A.); 2 Department of Prosthodontics, Faculty of Dentistry, Thamar University, Dhamar P.O. Box 13020, Yemen; m.n.alhajj@hotmail.com; 3 Department of Restorative Sciences (Prosthodontics), College of Dentistry, Kuwait University, Kuwait P.O. Box 24923, Kuwait

**Keywords:** lithium disilicate, nanoceramic, fracture load, CAD/CAM, dynamic loading

## Abstract

Fracture resistance is an important parameter used to predict the performance of indirect dental restorations. The purpose of this in vitro study was to assess the fracture load of posterior milled nanoceramic crowns, in comparison with the lithium disilicate crowns, after fatigue loading, for two different restoration occlusal thicknesses. Forty test metal dies were fabricated by duplicating a master metal model consisting of an anatomic abutment preparation of the maxillary first premolar for a single crown. The dies were divided into two groups of 20 each for the fabrication of nanoceramic (Lava Ultimate) and lithium disilicate (IPS e.max CAD) single crowns. Each material group was further divided into two sub-groups of 10 dies each, based on crown occlusal thickness, of 0.5-mm and 0.75-mm (*n* = 10). Dental Type V stone dies poured from polyvinyl siloxane impressions of the test metal dies were laboratory scanned in order to design and mill 40 ceramic crowns. The crowns were cemented on to the test metal dies with a self-adhesive resin luting cement. All crowns were thermocycled (2500 cycles) and mechanically loaded (250,000 cycles) in a chewing simulator followed by static loading until failure, and the values noted. The data were statistically analyzed by 2-way ANOVA and Tukey HSD post-hoc multiple comparison tests (α = 0.05). The mean fracture loads ranged from 1022 to 1322 N for nanoceramic crowns and from 1145 to 1441 N for the lithium disilicate crowns. Two-way ANOVA revealed insignificant differences between the nanoceramic and lithium disilicate crowns (*p* > 0.05) in terms of fracture load. Significant differences were noted in the fracture resistance of crowns based on occlusal thickness (303 N; *p* = 0.013) regardless of the material used. Multiple comparisons by Tukey HSD post-hoc test showed insignificant differences between the four material-occlusal thickness groups (*p* > 0.05). The nanoceramic crowns were found to be comparable with lithium disilicate crowns in terms of fracture load. The mean fracture loads of all of the tested crowns were within the normal physiological masticatory load limits. Based on the fracture-resistance results, nanoceramic crowns seem to be suitable for clinical use for the tested occlusal thicknesses.

## 1. Introduction

Hybrid ceramics have recently been developed for the computer-aided design and computer-aided-manufacturing (CAD-CAM) fabrication of indirect dental restorations, including complete-coverage crowns [1,2,3,4]. Resin nanoceramics (RNC) are a class of hybrid ceramics composed mainly of silica nanoparticles and zirconia or barium nanomers in an organic polymer matrix that is similar to composite resin [5]. The RNCs purportedly offer optimal fracture strength and flexibility through combining the characteristics of composite and ceramic materials [6]. Additionally, milled RNC definitive restorations do not require sintering, firing, or glazing for placement in the oral cavity [4,5,6]. Recent investigations examined the mechanical properties of CAD-CAM monolithic RNCs, including wear resistance [7,8], flexural strength [9,10], micro-tensile bond strength [11], marginal fit [12], and aesthetics [13] and found comparable or mixed results in relation to the traditional milled lithium disilicate and feldspathic ceramics. A current review [14] on the clinical outcomes of RNC single crowns found sparse data, reporting favorable short-term survival rates for single crowns, and observing restoration fracture as the main technical complication. Although several studies have analyzed the factors affecting the clinical performance of RNCs, evidence is scarce on the fracture resistance of posterior RNC indirect restorations, especially for complete contoured single crowns.

In the posterior area of the oral cavity, abutment height is often short after tooth preparation, thus limiting the inter-occlusal space available for the crown. Occlusal reduction of vital teeth is restricted in some situations due to excessive wear or in order to prevent pulpal exposure, which leads to minimal inter-occlusal restorative space. Ceramic materials with an ability to tolerate occlusal forces at reduced thicknesses without sustaining cracks or fractures are needed for such conditions. Suksuphan et al. [12] assessed the effects of different occlusal thicknesses (0.8 mm, 1 mm, and 1.5 mm) on the fracture resistance of molar nanoceramic crowns (filled with barium silica nanomers) and found insignificant differences. Zimmermann et al. [15], on the other hand, found significant differences between the fracture loads of molar RNC crowns of occlusal thicknesses of 0.5 mm, 1 mm, and 1.5 mm. In both studies [12,15], the crowns were fabricated using 4-axis milling machines. Another investigation [16] also examined the fracture load differences as a factor of RNC occlusal thickness, but with non-anatomical test specimens bonded to epoxy resin disks, and they reported no significant differences between the 0.5 mm, 1 mm, 1.5 mm, and 2 mm material thicknesses. Apart from these studies, the authors are unaware of any other evidence in the scientific literature that investigates the effect of occlusal thickness parameter on the fracture resistance of posterior RNC crowns manufactured by 5-axis milling, particularly with zirconia–silica-nanomer-infiltrated hybrid ceramics.

Studies have assessed the fracture resistance of monolithic molar lithium disilicate (LDS) complete-coverage crowns as a factor of occlusal thickness [15,17,18,19], finding significant [15], insignificant [17], or mixed results [18,19] in terms of fracture load differences. Yet, data is scarce on the fracture resistance of premolar LDS crowns with 0.5 mm minimal occlusal thickness.

The objective of this in vitro study was to determine and compare the fracture resistance of premolar complete-coverage crowns milled with monolithic resin nanoceramic versus monolithic lithium disilicate (control) for two different occlusal thicknesses, 0.5 mm and 0.75 mm, after thermomechanical aging. The null hypothesis was that there would be no differences in the fracture load of crowns for the two materials or for occlusal thicknesses, in terms of failure load values, after fatigue loading.

## 2. Materials and Methods

### 2.1. Sample Size Calculation

The sample size was calculated based on earlier related studies [12,15]. For an assumed mean difference of 685 newtons (N) and a standard deviation of 268 N [15], at α = 0.05 and observed power of 0.85 (G*Power v.3.1.9.7; Heinrich Heine University Düsseldorf, Düsseldorf, Germany), a required sample size of 10 crown specimens was determined for each crown occlusal thickness test group in order to estimate whether there was a significant difference in the fracture load.

### 2.2. Preparation of Reference and Test Die Models

**Figure 1:**
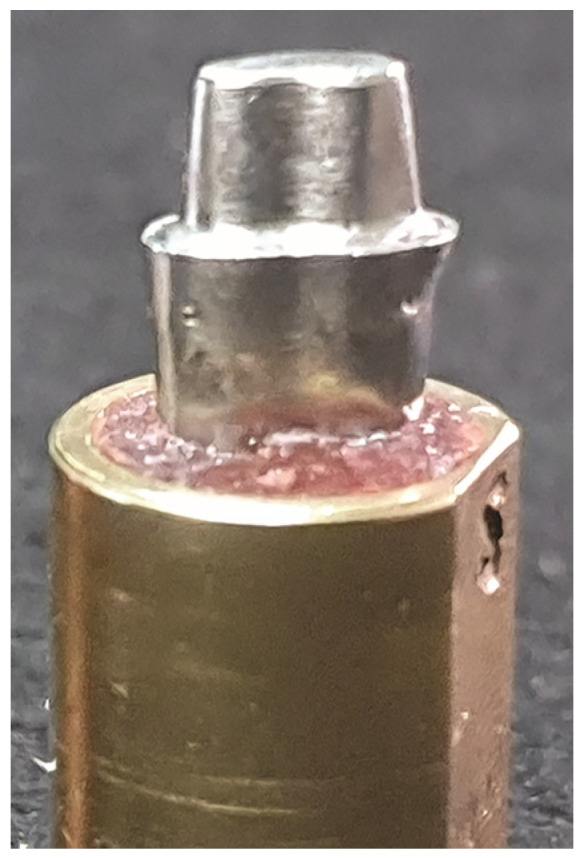
Reference metal die.

A cast-metal cobalt–chromium (Co-Cr) die (Remanium 800; Dentaurum GmbH & Co KG, Ispringen, Germany) derived from an ivorine maxillary second premolar tooth preparation (Typodont Tooth Columbia #4; Columbia Dentoform Corp., Long Island City, NY, USA) was used as a reference model in this study (Figure 1). The metal die had a 1-mm wide rounded shoulder margin, <20 degrees occlusal total angle of convergence, and 4 mm cervico-occlusal height, which was standardized using a tooth preparation guide, according to an earlier study [20].

**Figure 2:**
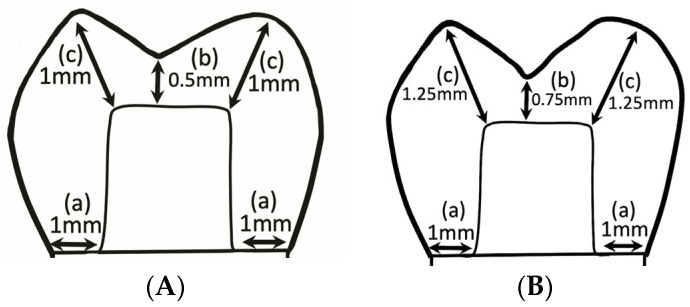
Schematic diagrams illustrating the crown design for 0.5–1 mm (**A**) and 0.75–1.25 mm (**B**) crown occlusal thicknesses. a. preparation marginal width; b. minimum occlusal thickness of crown; c. maximum occlusal thickness of crown.

Forty cast-metal Co-Cr test dies (Solidur CoCr; YETI Dentalprodukte GmbH, Engen, Germany) were fabricated by replicating the reference model. These dies were randomly assigned to one of the 2 crown groups: 20 to the RNC group (Lava Ultimate; 3M ESPE AG, Seefeld, Germany) and 20 to the LDS group (IPS e.max CAD; Ivoclar AG, Bad Säckingen, Germany). The 2 crown-material groups were further divided into 2 sub-groups based on a crown occlusal thickness of either 0.5 to 1 mm or 0.75 to 1.25 mm (*n* = 10) (Figure 2A,B). Next, 40 impressions (Express XT light body and regular body polyvinyl siloxane; 3M ESPE AG, Neuss, Germany) of the test metal dies were made with custom trays (Preci Tray; YETI Dentalprodukte GmbH, Engen, Germany) and poured into type V dental stone (Jade stone; Whip Mix Corp., Louisville, KY, USA). The dies were allowed to set for 24 h and were checked visually under a ×5 microscope (BM-1 Stereomicroscope; Meiji Techno Co., Ltd., Saitama, Japan) for voids and nodules, and they were matched with the corresponding metal dies if deemed satisfactory.

### 2.3. Digitization of Stone Dies, Milling, and Luting of Crown Specimens

The stone dies were digitized with a laboratory scanner (Medit T710; Medit Corp., Seoul, Republic of Korea) and the crowns were virtually designed on the digital dies with a software program (Dental CAD 3.0; Exocad GmbH, Darmstadt, Germany). A standardized occlusal anatomy was chosen for the maxillary second premolar with an average occlusal thickness of 0.5 mm at the central fossa and a maximum of 1 mm at the cusp area, and with 0.75 mm at the central fossa and a maximum of 1.25 mm at the cusp area for each crown-material group (*n* = 10). The cement space was set at 50 microns (µm) [12,21]. The monolithic crowns were milled (CEREC InLab MC X5, Dentsply Sirona Inc., Charlotte, NC, USA) using the data from LDS (IPS E.max CAD) and RNC blocks (Lava Ultimate). The RNC blocks contained 80 weight (wt.)% zirconia–silica nanomer and nanocluster fillers incorporated in a resin matrix (zirconia particle size 4 to 11 nanometers (nm); silica particle size 20 nm) [5,9,15]. The LDS crowns were crystallized (Programat P310; Ivoclar AG, Schaan, Liechtenstein) at 850 °C for 25 min. All of the crowns were washed and cleaned in an ultrasonic cleaner for 3 min [8] before undergoing finishing and polishing (Porcelain adjustment kit HP, Shofu finishing and polishing systems; Shofu Inc., Kyoto, Japan) on the stone dies. The crowns were assessed for fit on the assigned metal dies using United States Public Health Services (USPHS) and California Dental Association (CDA) criteria [21]. Once found satisfactory, the intaglio surface of the RNC and LDS crowns were sandblasted with 50 µm aluminum oxide (Al_2_O_3_) and treated with 4% Hydrofluoric acid (IPS ceramic gel, Ivoclar AG, Schaan, Liechtenstein) for 20 s, respectively, before being cemented onto the metal dies using a dual-cure self-adhesive luting cement (RelyX U200, 3M ESPE AG., Seefeld, Germany) by maintaining finger pressure for 2 min, followed by a constant static pressure load (22 N) for 5 min [21].

### 2.4. Thermomechanical Aging and Load-to-Failure Testing

**Figure 3:**
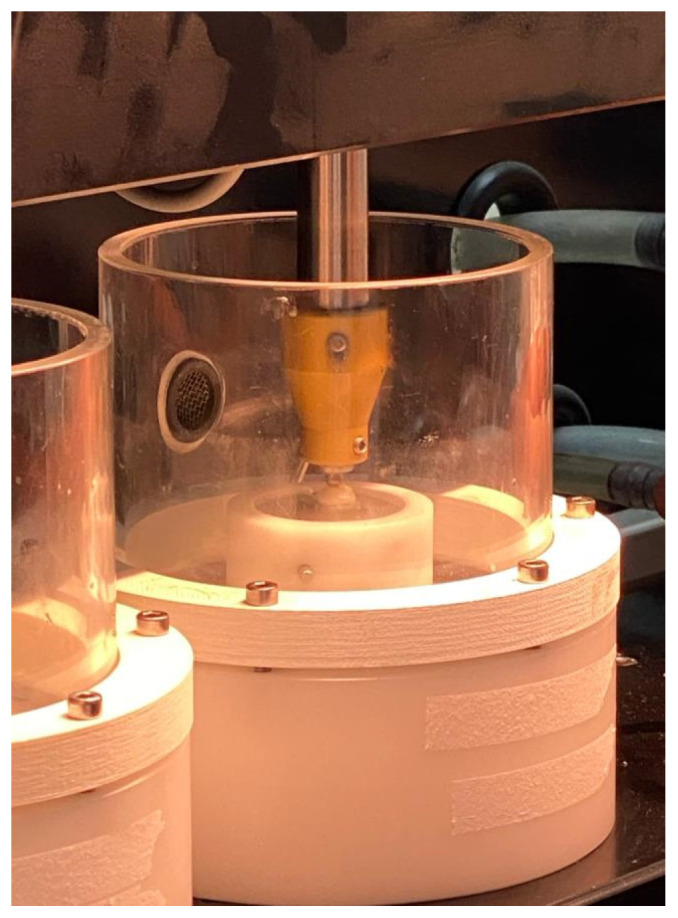
Thermocycling and mechanical loading of crowns using chewing simulator.

All crown specimens were fatigue loaded (250,000 cycles; 50 N; 1.25 Hertz [Hz]) [8,22] with a hemispherical antagonist in a chewing simulator (CS 4.4; SD Mechatronik GmbH., Bayern, Germany), by impacting the mesial triangular fossa with a vertical load, followed by a sliding horizontal movement, for each cycle (height, 2 mm; lateral movement, 0.7 mm) (Figure 3) [8,22]. Simultaneously, thermocycling of specimens in distilled water was completed (2500 cycles; 5 °C to 55 °C; dwell time 30 s).

**Figure 4:**
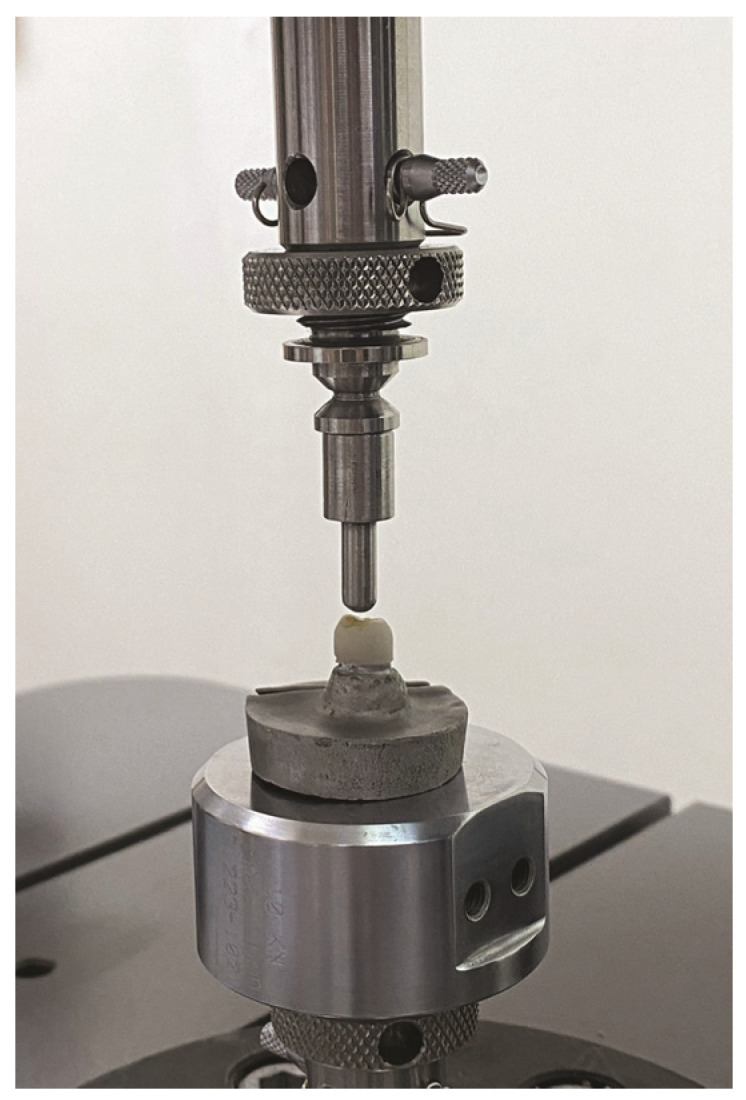
Compressive loading of crowns.

The specimens were inspected for cracks, chips, or other deformities after the cyclic loading phase. The 40 crown specimens were subjected to a static load (Instron ElectroPuls E3000; Instron Corp., Norwood, MA, USA) with a Ø4-mm hemispherical steel head indenter directed into the central fossa of the crown, at a crosshead speed of 0.5 mm per min, until failure (Figure 4) [8,22]. The fracture loads were measured using a software program (Bluehill V2.8; Instron Corp., Norwood, MA, USA).

The failed specimens were examine, and the failure modes were registered for each crown, based on the classification used in previous studies [9,20], as follows: type I, minimal chipping, with possibility of refinishing and repair; type II, loss of less than half of the crown; type III, crown fracture through midline with half the crown lost; and, type IV, severe fracture of the crown with a loss of more than half of the crown.

### 2.5. Statistical Analysis

Data analysis was completed using a statistical software program (IBM SPSS statistics, v28; IBM Corp., Armonk, NY, USA). Mean ± standard deviation (SD) values of fracture load were calculated for the different material and OT groups. The Shapiro–Wilk test was used to verify the normality of distribution of data and the Levene test was performed to check the homogeneity of variances. The data was found to be symmetric about the mean (*p* > 0.05). Descriptive statistics were performed for the test groups and the effects of material and occlusal thickness on the fracture load were statistically evaluated using 2-way analysis of variance (ANOVA) testing (α = 0.05). Tukey honestly significant difference (HSD) post-hoc multiple comparison tests were used to further assess the differences between the 4 material-occlusal thickness groups (α = 0.05).

## 3. Results

None of the crown samples showed cracks or fractures after undergoing the set TCML cycles, but all crowns failed during the static load test. The mean ± SD and median with inter-quartile range (IQR) fracture-load values of RNC and LDS crown groups are presented in Table 1.

**Figure 5:**
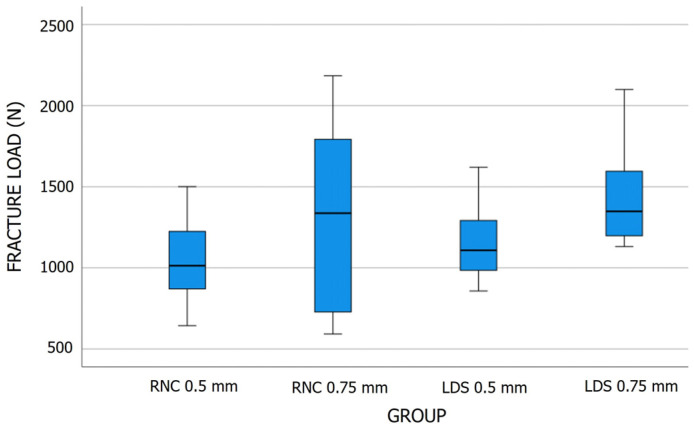
Box plot graph showing distribution of fracture load (N) across different material-occlusal thickness groups. LDS, lithium disilicate; N, Newtons; RNC, resin nanoceramic.

The box plot (Figure 5) shows the fracture-load data for the different material-occlusal thickness groups for five statistics: minimum, first quartile, median, third quartile, and maximum.

The 2-way ANOVA tests revealed significant differences in the fracture load between the crown groups based on occlusal thickness (*p* = 0.013); however, the two materials did not exhibit any significant differences, regardless of the occlusal thickness (*p* > 0.05) (Table 2). The interactions between the material and occlusal thickness were found to be insignificant (*p* > 0.05). Tukey HSD post-hoc multiple comparison testing confirmed insignificant fracture load differences between the crown groups with different material-occlusal thickness combinations (*p* > 0.05). 

All crown fractures were brittle, occurring vertically or diagonally from the occlusal surface and involving the full thickness of ceramic. The spread of the RNC and LDS crown-failure types are shown in Table 3.

**Figure 6:**
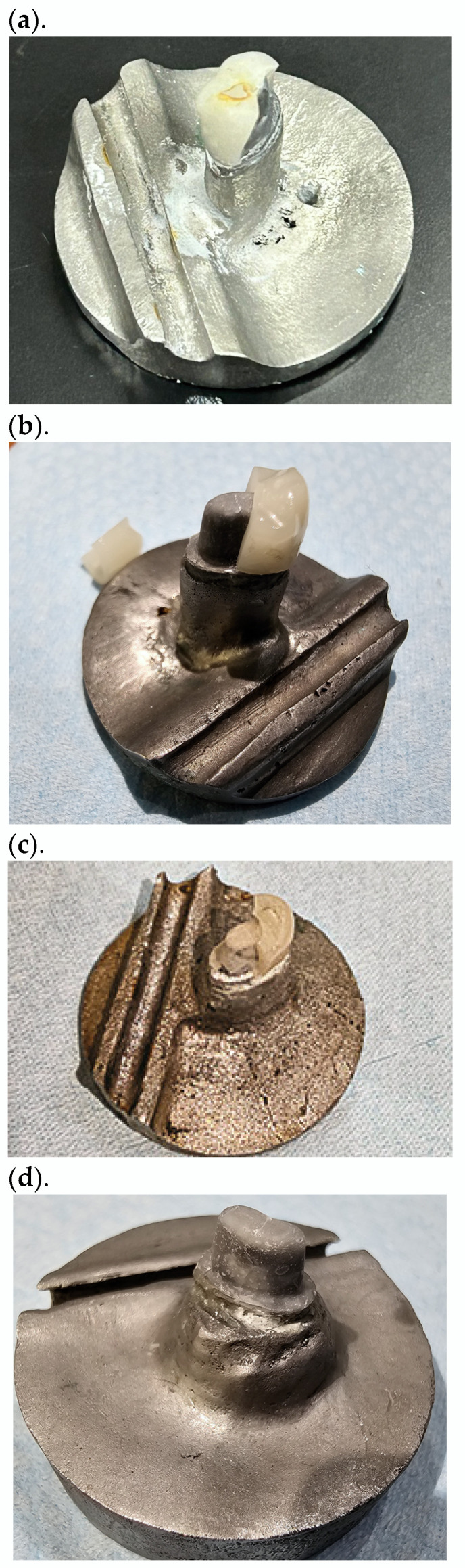
Occlusal view of representative specimen. (**a**) Crown type II failure, loss of less than half crown; (**b**) crown type III failure, crown fracture through midline with half-crown loss; (**c**) crown type IV failure, severe fracture of crown; and (**d**) crown type IV failure, severe fracture with loss of entire crown.

None of the crown samples failed with minor cracks or fractures when subjected to compressive loading. A part or complete crown always fractured in this study. Figure 6a–d are representative samples of the different failure types recorded in this study.

## 4. Discussion

In this in vitro study, the mean fracture-resistance values of monolithic RNC crowns were compared with those of LDS crowns and found to be insignificant. Also, the paired comparisons between the different material-occlusal thickness groups for fracture loads did not identify significant differences, consequently affirming the null hypothesis. The significant differences in the failure load values between the crowns with different occlusal thicknesses (0.5 mm and 0.75 mm) allowed rejection of this part of the null hypothesis.

The mean fracture-load values (1022 to 1332 N) of RNC crowns in this study were relatable to the values reported in a recent investigation [15] on molar nanoceramic crowns (655 to 1170 N) having 0.5 to 1 mm occlusal thickness, although the lower side of the value range was much higher in the current study. Conversely, another study [12] found that molar RNC crowns with 0.8 to 1 mm occlusal thickness resisted fracture at a 2000 N static load. The potential reasons for the disparity in results between the current and past studies [12,15] could relate to the differences in the abutment die material, cement space parameters, type of antagonistic element used, number of mechanical loading cycles, RNC composition, CAD-CAM systems used, crown design, crown type, and luting cements used. The previous investigators [12,15] had adhesively luted the crowns onto additive manufactured-methacrylate-abutment dies, whereas metal dies were used in this study. The crowns were cyclically loaded for 1.2 million cycles by Zimmermann et al. [15], whereas Suksuphan et al. [12] did not fatigue load the crowns before fracture loading, which might explain the survival of the 0.8 mm and 1 mm thick crowns under a 2000 N compressive load. Additionally, the molar crowns were designed with uniform thickness on the occlusal surface in the earlier studies [12,15], as compared with the present study, where the premolar crown thickness varied between the central fossa and cuspal regions.

The mean fracture loads of LDS crowns (1141 to 1445 N) in this study were in agreement with Chen et al. [17], who reported values in the range of 1228 to 1377 N for monolithic LDS crowns of 0.7 mm occlusal thickness, The current values were also within the range of fracture loads (1054 to 1752 N) for LDS crowns of 1 mm occlusal thickness that was recorded by Nawafleh et al. [19] both with and without fatigue loading. The results, however, did not concur with two studies [15,18], which reported fractures with loads from 838 to 1027 N for 0.8 to 1 mm thick crowns. The likely causes for the discrepancies in values could be related to the fatigue-loading parameters applied (1.2 million and 5 million cycles) before fracture testing [15,18], apart from the use of digitally printed resin abutment dies for the cementation of crowns. The surface treatment and the luting cement used might additionally have played roles in the fracture load differences.

The present study did not find significant differences in fracture load between the RNC and LDS crowns, which is in agreement with Zimmerman et al. [15] and Güleç and Sarıkaya [22]. The findings, however, conflicted with other studies [8,9,23], which found significant differences between the RNC and LDS crowns, and between other hybrid ceramic PICN and LDS crowns. These differences in fracture load could be attributed to the lack of fatigue loading [9], crown occlusal thicknesses of 1.5 mm [8] or 2 mm [9], the type of material [23], the finish line preparation design [23], and the use of different RNC materials for crown fabrication [8,9].

In the current study, the RNC 0.75 mm crowns demonstrated the highest fracture loads (2184 N) and median (1338 N) values among all of the material-occlusal thickness groups; however, its inter-quartile range was also much larger than for all other groups (Table 1), which was due to some crowns fracturing under 1000 N load. These outcomes might have affected the significance of the results, particularly in relation to the fracture-load differences found between the 0.5 mm and 0.75 mm crowns. All crowns tested (RNC and LDS) in this investigation, regardless of occlusal thickness, demonstrated mean and median fracture loads exceeding the average clinical masticatory forces (600 to 900 N) in the posterior region [24].

None of the crowns de-bonded, cracked, or fractured after thermomechanical loading in this study, which is similar to the results of earlier investigations on RNC [8,15] and LDS crowns [19]. Failure of one or more LDS crowns of occlusal thickness 0.5 to 1 mm by fatigue loading has been documented previously [15,18], although it is to be noted that the crowns in those studies were subjected to 1.2 million cycles at 50 N [15], and 5 million loading cycles at 275 N [18]. The current study pre-loaded the crowns to 250,000 cycles, which is equivalent to 1 year of clinical service. Outcomes might have differed with higher mechanical loading cycles. It is important to emphasize, however, that all reported fatigue-loading failures occurred with LDS crowns and none occurred with RNC crowns, regardless of occlusal thickness [15].

The failure pattern of LDS crowns was consistent with the observations of recent studies in that the fractures were brittle and complete, involving the full thickness of the ceramic [9,19,21]. The RNC-crown-failure types were like those of the LDS crowns except that there were fewer type IV severe fractures (Table 3). The RNC-crown-failure characteristics were inconsistent with those from an earlier study [9], where minor chipping (type I failure) was observed with 70% of the tested crowns, at a mean failure load of 1562 N. The current investigation did not elicit any type I failures. The likely causes for the type 1-failure-mode presentation in the previous study [9] were the increased occlusal thickness of 2 mm, different abutment substrate usage, and the particular RNC material used. The RNC-crown-failure characteristics could not be compared with other crown-occlusal-thickness-related studies, as one of the authors’ Suksuphan et al. [12] reported no failures at a 2000 N static load, and Zimmermann et al. [15] did not describe the crown failure. The majority of the failures that were recorded with LDS crowns (11 of 20) were of the type IV category (severe fracture of the crown) (Table 3), thus closely matching the earlier published data in which more than 75% of zirconia–lithium silicate-crown failures were of type IV [9,21]. The differences in the failure patterns of RNC versus LDS crowns might be due to the difference in the elastic moduli of the two materials (RNC, 15 GPa; LDS, 95 GPa) (Table 3) [15].

A dual-cure self-adhesive resin cement was used for luting the crowns on the metal dies in this study. Results could have been different if a dual-cure adhesive resin cement was used with different surface-treatment protocols, although a recent study has shown no effect of using either cement on the fracture resistance of nanoceramics [10]. All previous studies [12,15,17,18] exploring the effects of occlusal thickness on fracture load had used 4-axis milling machines, except for that of Nawafleh et al. [19], which assessed LDS crowns. The crowns in this investigation were machined with a 5-axis milling unit, which might have affected the fracture-load outcomes.

In this study, 0.75–1.25 mm occlusal thickness was used as the control group for the two crown materials, based on the findings of previous studies, where 0.8 mm RNC [12] and 0.7 mm LDS crowns [17] were found to be comparable with the more traditional crown thicknesses of 1.25–1.5 mm in terms of fracture loads [12,17]. Additionally, the crown design of the control group was closer to the clinical reality, where the central fossa region is often found to be thinner than the cuspal regions. Nevertheless, comparison of the current 0.5 mm occlusal thickness groups with 1–1.25 mm minimal = –thickness crowns would have revealed more information on the fatigue and fracture behavior of the tested materials.

There are some probable limitations in this study warranting discussion. Firstly, the crowns were compressively loaded to failure on metal dies in this study, following the study design used in earlier investigations [21,25]. The use of epoxy-resin or composite-resin abutments with elastic moduli values close to human dentine may have replicated the oral conditions better. Past studies [18,19,21] have indicated some disadvantages with using natural teeth, as they may be more susceptible to fracture under static loading, apart from the issue of standardization for comparison purposes. It needs to be noted, however, that a recent study [17] found no significant differences between the fracture loads of monolithic lithium disilicate ceramic crowns when abutment substrates of different elastic moduli (with 80 GPa difference) were used. Secondly, in this investigation the specimens were subjected to 250,000 loading cycles, which is equivalent to more than 1 year of masticatory simulation in the mouth [9,10,12]. The findings might have been different if a greater number of fatigue cycles were applied. However, it is worth mentioning that recent studies [8,22] documented no differences in the fracture resistance of RNC versus LDS crowns over 1.2 million cycles and without fatigue loading. Another investigation also found no fracture-load differences before or after thermomechanical aging (500,000 cycles), although the crowns examined were made of zirconia lithium silicate (ZLS) [21].

Future studies are required to analyze the fracture load of additively-manufactured hybrid-nanoceramic crowns, with different abutment heights and longer artificial aging periods, to analyze their performance in comparison with the milled-nanoceramic variants. Investigations comparing the fracture resistance of crowns made from monolithic translucent (5% yttria tetragonal zirconia polycrystal) 5Y-TZP, fully crystallized ZLS, heat-pressed ZLS, Bio high performance polymer (BioHPP) [26], or polymer-infiltrated ceramic network (PICN) with other nanoceramic materials are necessary to test the strength of the new hybrid ceramics further. Prospective clinical studies assessing the outcomes of posterior nanoceramic crowns in relation to the traditional glass or zirconia ceramics will aid in affirming the outcomes of the current study.

## 5. Conclusions

Based on the results of this in vitro study, the following conclusions were reached:

i. There were no significant differences in fracture load between the two tested materials (nanoceramic and lithium disilicate).

ii. Occlusal thickness influenced the fracture load, regardless of the material used; however, the comparisons between material and occlusal thickness showed no differences.

iii. The mean fracture-load values of posterior nanoceramic crowns were within the normal bite-force limits.

## Figures and Tables

**Table 1:** Fracture load of monolithic RNC and LDS crowns (N), (*n* = 10). IQR, inter-quartile range; LDS, lithium disilicate; OT, occlusal thickness; RNC, resin nanoceramic; SD, standard deviation.

Material	Mean ± SD	Minimum	Maximum	Median	IQR
RNC—OT 0.5 mm	1022 ± 253	644	1501	1014	386
RNC—OT 0.75 mm	1332 ± 566	592	2184	1338	1091
LDS—OT 0.5 mm	1145 ± 237	858	1621	1109	353
LDS—OT 0.75 mm	1441 ± 306	569	2100	1131	216

**Table 2:** Two-Way ANOVA results for fracture loading of RNC and LDS crowns. ANOVA, Analysis of variance; LDS, lithium disilicate; RNC, resin nanoceramic.

Variables of Interest	Type III Sum of Squares	Df	Mean Square	F	*p*
Material LDS RNC	134,212.23	1	134,212.23	1.01	0.323
Occlusal thickness (OT) 0.5 mm 0.75 mm	918,393.03	1	918,393.03	6.88	**0.013**
Material × Occlusal thickness (OT)	600.63	1	600.63	0.01	0.947

**Table 3:** RNC and LDS crown-failure pattern. Type I, minimal chipping, with possibility of refinishing and repair; Type II, loss of less than half of the crown; Type III, crown fracture through midline with half the crown lost; and, Type IV, severe fracture of the crown with loss of more than half the crown; LDS, lithium disilicate; RNC, resin nanoceramic.

Material	Type I	Type II	Type III	Type IV
RNC	0	8	6	6
LDS	0	4	5	11

## Data Availability

The raw data supporting the conclusions of this article will be made available by the authors on request.
